# Comorbidity Risk Assessment in Medicare Beneficiaries with Neurodevelopmental Disorders

**DOI:** 10.1093/geroni/igaf122.3735

**Published:** 2025-12-31

**Authors:** Bianca Rodriguez, Allison Willis

**Affiliations:** Perelman School of Medicine, Philadelphia, Pennsylvania, United States; Perelman School of Medicine, Philadelphia, Pennsylvania, United States

## Abstract

Neurodevelopmental disorders (NDDs) are lifelong conditions that affect brain development and functioning, with varying levels of impairment on daily life. Advances in technology have extended life expectancy for individuals with NDDs; however, little is known about their health in older adulthood. Using a 20% random sample of Medicare beneficiaries in 2021, we identified adults aged 65 and older with autism spectrum disorder, cerebral palsy, muscular dystrophy, or spina bifida. Demographic characteristics and comorbidities were compared to a control group without NDDs. Compared with controls, older adults with NDDs were younger on entry into Medicare, had distinct sex distributions, and were less racially and ethnically diverse. Neurological conditions—including epilepsy, multiple sclerosis, and migraine—were markedly more prevalent among NDD groups, as were psychiatric disorders such as anxiety, depression, and psychosis. Substance use disorders, particularly alcohol abuse and dependence, occurred at nearly twice the rate of controls. Vascular conditions, including myocardial infarction, atrial fibrillation, heart failure, ischemic heart disease, and stroke, were also elevated, averaging twofold higher in NDD groups. Neurodegenerative diseases, notably Alzheimer’s dementia and Parkinson’s disease, were up to three times more common among individuals aging with NDDs, underscoring the dual impact of lifelong neurodevelopmental conditions and aging-related decline. These findings demonstrate that while vascular and substance use disorders contribute to the excess burden of disease, the most pronounced disparities lie in neurological, psychiatric, and neurodegenerative comorbidities. This highlights the urgent need for research on neurodegeneration risk in NDD populations and for healthcare adaptations to better support individuals with lifelong NDDs.

